# Supplementary material for: Association of Metabolomic Change and Treatment Response in Patients with Non-Alcoholic Fatty Liver Disease
Source: Biomedicines. 2022 May 24;10(6):1216. doi: 10.3390/biomedicines10061216 (PMC9220113; doi:10.3390/biomedicines10061216)

Supplementary material

Table of contents

1. Supplementary Table S1
2. Supplementary Table S2
3. Supplementary Table S3
4. Supplementary Figure S1
5. Supplementary Figure S2

Supplementary Table S1. Significantly different changes in metabolite levels between the good and non-response groups were defined by transient elastography after receiving two treatments (ezetimibe + statin and statin alone). (a) Significant metabolites associated with an improvement in CAP values. (b) Significant metabolites associated with an improvement in LSM values. Metabolite changes were defined as changes in concentration compared with the baseline values. A *P* value of <0.05 was considered significant, and the FDR *P* value of <0.1.

(a) Steatosis	Metabolite	% change from the baseline		*P* value	FDR *P* value
		Good	Poor		
Ezetimibe + statin (n = 18)	TG 17:1_32:1	−81.7 [−93.3 to −76.6]	−25.6 [−62.2 to +13.0]	0.001	0.10
	TG 18:0_32:1	−71.7 [−73.8 to −69.8]	−42.8 [−54.3 to +76.4]	0.002	0.10
	TG 16:0_30:2	−85.8 [−95.8 to −76.2]	−52.0 [−65.1 to +136.9]	0.003	0.10
	TG 16:1_32:2	−70.4 [−94.6 to −60.0]	−15.1 [−40.8 to +24.3]	0.003	0.10
	TG 16:1_34:0	−66.0 [−77.8 to −63.3]	−23.0 [−55.6 to +22.0]	0.003	0.10
	TG 16:0_34:1	−66.2 [−75.0 to −60.1]	−40.8 [−56.2 to −2.4]	0.005	0.10
	TG 16:1_34:1	−63.2 [−81.5 to −53.7]	−22.7 [−50.8 to +16.5]	0.005	0.10
	TG 20:4_32:1	−70.5 [−74.3 to −67.7]	−23.1 [−45.1 to +94.9]	0.005	0.10
	TG 22:6_32:0	−74.6 [−83.3 to −64.7]	−50.1 [−62.3 to −30.2]	0.005	0.10
	TG 14:0_38:5	−73.8 [−85.7 to −62.6]	−5.6 [−45.6 to +66.9]	0.007	0.10
	TG 16:0_32:0	−80.4 [−89.9 to −61.7]	−30.7 [−56.7 to +21.9]	0.007	0.10
	TG 16:0_32:1	−80.9 [−86.3 to −68.1]	−54.3 [−66.0 to +53.5]	0.007	0.10
	TG 16:1_32:1	−71.6 [−80.5 to −65.8]	−29.5 [−51.3 to +8.5]	0.007	0.10
	TG 18:1_30:0	−76.9 [−88.6 to −58.8]	−49.1 [−62.4 to +61.5]	0.007	0.10
	Saturated_mono−unsaturated TG	−67.9 [−78.9 to −58.2]	−42.6 [−58.2 to +14.1]	0.01	0.07
	Xanthine	−39.7 [−62.3 to −14.4]	+47.0 [−7.3 to +80.7]	0.01	0.03

Statin (n = 23)	FA 18:1	−45.7 [−60.9 to −29.2]	+35.4 [−10.8 to +54.5]	<0.001	0.003
	FA 18:2	−43.5 [−61.6 to −17.0]	+26.5 [−0.2 to +41.8]	0.001	0.003
	C2	−21.6 [−30.2 to −15.8]	+5.4 [−12.3 to +27.2]	0.002	0.03
	DHA	−34.4 [−51.6 to −14.6]	+21.5 [−13.3 to +91.2]	0.008	0.02
	DG 16:1_18:2	−56.7 [−69.1 to −51.4]	−25.8 [−44.9 to +8.8]	0.02	0.08
	DG 16:1_18:1	−59.4 [−68.7 to −41.4]	−26.9 [−50.4 to −6.7]	0.02	0.08

**(b) Stiffness**	**Metabolite**	**% change from the baseline**		***P* value**	**FDR *P* value**
		**Good**	**Poor**		
Ezetimibe + statin (n = 20)	Choline	+8.2 [−8.1 to +32.9]	−17.2 [−25.1 to −2.4]	0.05	0.05
Statin (n = 19)	Hippuric acid	+12.1 [−12.9 to +123.0]	−33.1 [−53.4 to −24.6]	0.005	0.02
	Hypoxanthine	−77.7 [−89.7 to +64.7]	+69.4 [+31.6 to +259.8]	0.02	0.04

Supplementary Table S2. All significantly different changes in metabolite levels (p-value < 0.05 without multiple comparisons) between the good and non-response groups according to the MRI-PDFF values after receiving two treatments (ezetimibe + statin and statin alone), (a) in the ezetimibe + statin group and (b) in the statin-only group

**(a) Metabolite**	**% change from the baseline**		***P* value**	**FDR *P* value**
	**Good**	**Poor**		
TG 16:1_33:1	−46.1 [−75.9 to −29.4]	+8.5 [−30.1 to +43.3]	0.003	0.40
PC ae C32:2	−15.4 [−27.8 to −13.1]	−31.3 [−41.1 to −27.6]	0.006	0.39
TG 16:0_30:2	−75.5 [−85.8 to −50.1]	−47.0 [−65.1 to +62.3]	0.006	0.40
Xanthine	−21.4 [−60.2 to +10.6]	+19.5 [−7.3 to +64.9]	0.01	0.02
TG 16:1_32:2	−52.5 [−72.6 to −16.9]	−5.0 [−32.2 to +25.1]	0.01	0.40
TG 22:6_32:0	−65.5 [−80.2 to −54.4]	−49.3 [−56.0 to −15.9]	0.01	0.40
TG 22:5_32:0	−72.2 [−82.2 to −46.6]	−42.7 [−61.4 to −6.3]	0.02	0.40
TG 17:1_32:1	−61.0 [−79.5 to −33.5]	−19.2 [−48.5 to +16.4]	0.02	0.40
TG 18:0_32:2	−72.5 [−87.9 to −42.4]	+4.2 [−71.8 to +63.5]	0.02	0.40
PC aa C42:1	0.0 [−19.8 to +7.5]	−27.0 [−34.5 to −10.2]	0.02	0.63
TG 14:0_38:5	−48.6 [−70.3 to −15.8]	−2.0 [−28.2 to +54.0]	0.02	0.40
TG 16:1_34:0	−60.3 [−72.3 to −28.0]	−15.3 [−52.7 to +22.0]	0.02	0.40
TG 18:1_30:0	−61.7 [−81.0 to −51.0]	−48.8 [−61.4 to +23.1]	0.02	0.40
TG 18:0_32:1	−63.3 [−73.0 to −42.0]	−40.0 [−53.4 to +48.0]	0.03	0.40
Homocysteine	+8.6 [−14.0 to +33.1]	−19.9 [−26.8 to −4.4]	0.03	0.33
3-Met-His	+300.8 [+50.5 to +1141.6]	+22.9 [−38.8 to +75.5]	0.03	0.33
TG 16:0_32:0	−55.7 [−80.4 to −26.2]	−26.3 [−41.2 to +8.8]	0.03	0.40
TG 18:1_30:1	−54.1 [−72.6 to −22.4]	−16.1 [−47.1 to +51.5]	0.03	0.40
Hex2Cer(d18:1/16:0)	−23.5 [−35.4 to −11.2]	−39.7 [−46.8 to −24.1]	0.04	0.34
TG 16:0_34:1	−59.4 [−70.2 to −35.9]	−38.9 [−54.3 to −2.4]	0.04	0.40
Cer(d18:2/16:0)	−18.3 [−32.0 to +11.5]	22.8 [−7.8 to +62.4]	0.04	0.55
PC aa C34:4	−30.1 [−47.8 to −14.5]	−9.1 [−35.3 to +2.8]	0.04	0.63
TG 14:0_34:1	−64.5 [−83.8 to −46.2]	−50.7 [−62.2 to +21.0]	0.04	0.40
TG 16:0_33:1	−59.4 [−75.3 to −26.8]	−27.2 [−60.2 to +27.9]	0.05	0.40
TG 16:0_34:0	−50.7 [−67.0 to −27.6]	−26.5 [−43.7 to −0.6]	0.05	0.40
PC ae C32:1	−26.9 [−35.9 to −12.3]	−38.0 [−40.3 to −32.7]	0.05	0.63
PC ae C44:6	+7.5 [−19.4 to +18.1]	−16.2 [−20.4 to −9.5]	0.05	0.63
Hex2Cer(d18:1/22:0)	−21.6 [−37.0 to −8.7]	−45.5 [−61.7 to −22.7]	0.05	0.34
Saturated_mono-unsaturated TG	−58.0 [−71.3 to −38.6]	−39.6 [−55.7 to +8.4]	0.05	0.35
TG 14:0_36:1	−49.7 [−74.3 to −26.0]	−29.5 [−49.2 to +26.9]	0.05	0.40

(b) Metabolite	% change from the baseline		*P* value	FDR *P* value
	Good	Poor		
TG 20:5_36:2	−43.5 [−51.1 to −26.5]	+14.2 [−15.0 to +92.3]	<0.001	0.04
TG 18:1_38:6	−53.2 [−63.2 to −42.4]	−13.8 [−37.2 to +23.3]	<0.001	0.04
C2	−22.2 [−30.0 to −17.1]	+5.4 [−12.3 to +29.5]	0.001	0.007
FA 18:2	−39.0 [−60.3 to −6.3]	+8.6 [−18.1 to +40.0]	0.001	0.009
TG 16:0_40:6	−47.1 [−61.6 to −39.6]	−32.8 [−38.8 to −6.1]	0.002	0.15
FA 18:1	−42.9 [−51.4 to −19.1]	+15.8 [−28.1 to +53.0]	0.003	0.009
TG 16:0_40:8	−57.7 [−70.6 to −26.2]	−17.3 [−38.1 to +9.8]	0.003	0.17
TG 18:2_38:6	−56.5 [−64.0 to −40.2]	−24.2 [−38.1 to −10.6]	0.006	0.24
TG 16:0_40:7	−50.1 [−66.1 to −35.7]	−7.7 [−36.7 to +20.6]	0.008	0.28
DHA	−36.1 [−46.0 to −17.4]	+13.3 [−20.5 to +78.2]	0.009	0.02
TG 22:6_34:1	−58.1 [−62.3 to −29.7]	−18.8 [−39.1 to +7.4]	0.009	0.25
TG 22:6_34:2	−48.6 [−67.9 to −26.1]	−19.2 [−33.5 to +8.6]	0.01	0.28
TG 22:5_34:2	−57.0 [−64.7 to −30.5]	−26.6 [−42.9 to −6.4]	0.01	0.28
TG 18:1_38:7	−38.7 [−53.2 to +2.7]	+11.9 [−4.6 to +45.3]	0.02	0.28
TG 20:5_36:3	−22.8 [−57.8 to +8.0]	+46.5 [+1.5 to +104.0]	0.02	0.28
TG 18:2_38:5	−49.0 [−54.6 to −25.6]	−9.5 [−32.6 to +14.8]	0.02	0.29
PC aa C42:1	−27.5 [−32.8 to −16.2]	−14.0 [−21.7 to −1.2]	0.02	0.51
PC aa C38:6	−37.7 [−43.3 to −11.4]	−20.7 [−30.7 to +3.1]	0.02	0.51
PC aa C40:6	−43.8 [−50.9 to −6.2]	−17.5 [−30.3 to −7.7]	0.02	0.51
TG 22:6_32:0	−72.1 [−81.6 to −54.5]	−47.9 [−67.1 to −15.0]	0.02	0.37
TG 20:5_34:2	−52.4 [−66.8 to −26.4]	−15.3 [−44.8 to +26.1]	0.03	0.41
TG 22:5_34:1	−44.6 [−64.5 to −32.0]	−29.6 [−44.3 to +0.9]	0.03	0.41
TG 20:0_32:3	−62.4 [−67.6 to −25.9]	−27.3 [−44.7 to +5.0]	0.03	0.41
TG 20:1_32:3	−61.7 [−79.3 to −32.1]	−4.6 [−57.2 to +49.9]	0.04	0.43
PC aa C38:0	−36.9 [−42.1 to −25.2]	−23.4 [−31.5 to −8.6]	0.04	0.58
TG 18:3_36:4	−51.2 [−70.8 to −28.3]	−15.6 [−51.1 to +14.0]	0.04	0.43
TG 18:3_35:2	−47.5 [−71.0 to −38.2]	−28.8 [−43.3 to +33.1]	0.04	0.43
TG 18:1_36:5	−45.4 [−63.4 to −19.9]	−27.7 [−40.4 to +9.8]	0.05	0.43
DG 16:1_18:2	−56.6 [−65.4 to −35.3]	−26.2 [−44.9 to +5.6]	0.05	0.38
TG 22:6_32:1	−65.6 [−71.6 to −36.8]	−36.3 [−56.6 to −26.2]	0.05	0.43
TG 20:0_32:4	−65.5 [−67.5 to −34.6]	−28.4 [−43.3 to +8.9]	0.05	0.43
TG 22:6_34:3	−54.7 [−70.4 to −36.1]	−37.4 [−48.1 to −10.8]	0.05	0.43
PC aa C38:5	−14.1 [−45.0 to −5.2]	−1.2 [−15.1 to +6.3]	0.05	0.58

Supplementary Table S3. All significant baseline metabolite levels (Wilcoxon's test, P value of <0.05 was considered significant, and the FDR P value of <0.1) between the good and non-response groups according to the MRI-PDFF values after receiving two treatments (ezetimibe + statin and statin alone), (a) in the ezetimibe + statin group and (b) in the statin-only group

**(a) Metabolite**	**Baseline concentration (μM) [Q1-Q3]**		***P* value**	**FDR *P* value**
	**Good**	**Poor**		
Hex3Cer(d18:1/16:0)	0.98 [0.83 to 1.17]	1.36 [1.19 to 1.45]	0.001	0.03

**(b) Metabolite**	**Baseline concentration (μM) [Q1-Q3]**		***P* value**	**FDR *P* value**
	**Good**	**Poor**		
Ornithine	95.0 [72.2 to 113.8]	141.4 [125.5 to 172.2]	<0.001	0.007
FA 18:2	60.8 [56.3 to 73.3]	41.3 [37.7 to 62.4]	0.01	0.04
FA 18:1	251.7 [206.8 to 308.2]	165.7 [127.0 to 230.9]	0.01	0.04
Methionine sulfoxide	0.64 [0.48 to 0.86]	0.97 [0.76 to 1.12]	0.01	0.09
Homocysteine	3.94 [3.08 to 5.10]	5.33 [4.35 to 6.84]	0.01	0.09
Kynurenine	1.77 [1.45 to 2.14]	2.28 [1.98 to 2.53]	0.02	0.09
DG 18:2_18:2	9.79 [9.00 to 15.07]	6.82 [4.56 to 9.08]	0.02	0.099
DG 16:1_18:2	1.93 [1.53 to 2.06]	1.45 [1.04 to 1.78]	0.02	0.099

Supplementary Figure S1. Study design

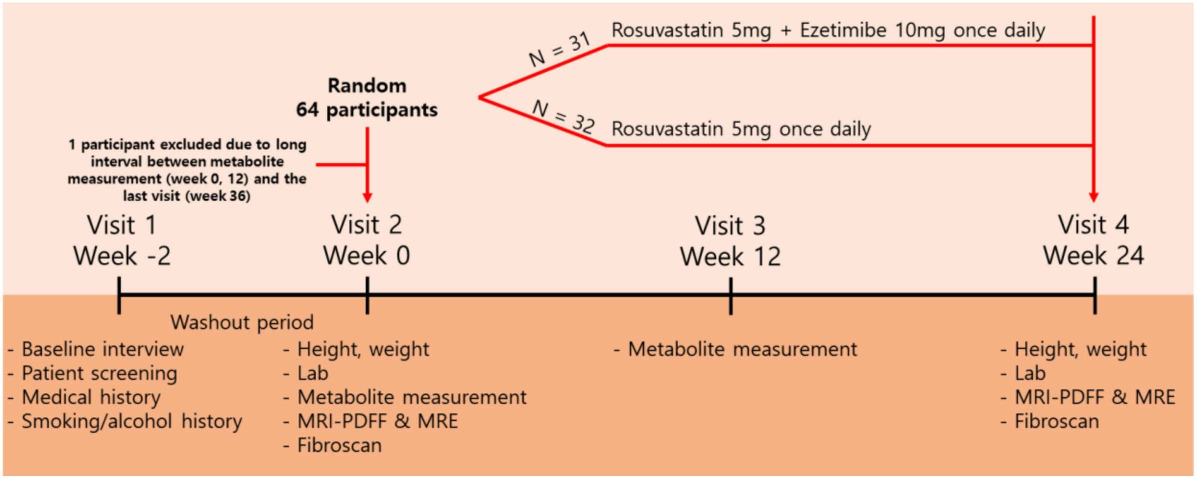

Supplementary Figure S2. Box plots of the % change in metabolite levels from baseline concentration between the two treatment groups. Significant changes in metabolite levels between the good and non-responders, liver fat measured by (a) MRI-PDFF, and liver stiffness measured by (b) MRE

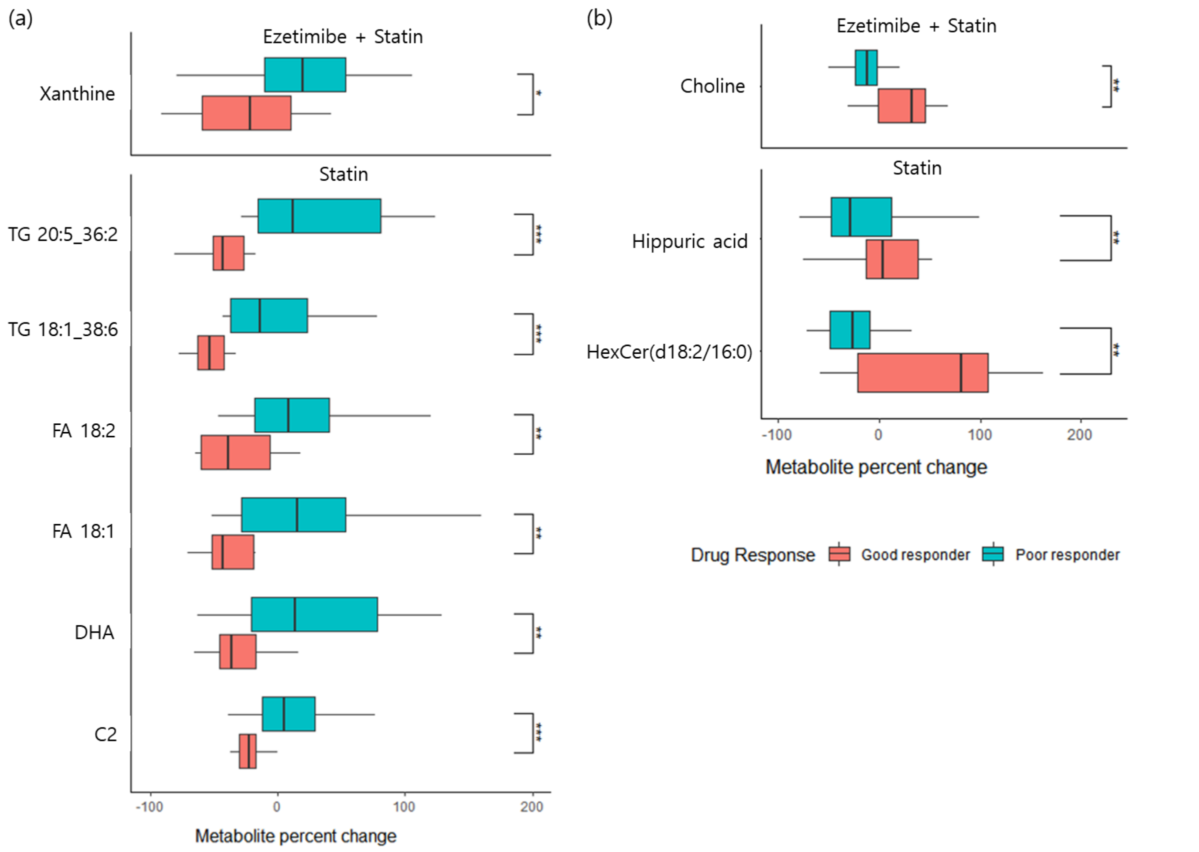

Supplement: Supplementary file 1 [file biomedicines-10-01216-s001.zip › biomedicines-1737079-supplementary.pdf]
